# Complete thoracoscopic S9 and/or S10 segmentectomy through a pulmonary ligament approach: a retrospective study

**DOI:** 10.1186/s13019-023-02256-8

**Published:** 2023-04-17

**Authors:** Shota Mitsuboshi, Takako Matsumoto, Motoka Omata, Hiroaki Shidei, Akira Ogihara, Akihiro Koen, Hiroe Aoshima, Tamami Isaka, Masato Kanzaki

**Affiliations:** The Department of Thoracic Surgery, Tokyo Women’s Medical University, 8-1 Kawada-cho, Shinjuku-ku, Tokyo, 162-8666 Japan

**Keywords:** Pulmonary ligament, Segmentectomy, Lung cancer, Thoracoscopic surgery, Lower lobe

## Abstract

**Background:**

The high resolution of computed tomography has found the pulmonary ligaments that consists of a double serous layer of visceral pleura, forms the intersegmental septum, and enters the lung parenchyma. This study aimed to investigate the clinical feasibility of thoracoscopic segmentectomy (TS) of the lateral basal segment (S9), posterior basal segment (S10), and both through the pulmonary ligament (PL).

**Methods:**

Between February 2009 and November 2021, 542 patients underwent segmentectomy for malignant lung tumors at Tokyo Women’s Medical University Hospital (Tokyo, Japan). This study included 51 patients. Among them, 40 underwent a complete TS of the S9, S10, or both by the PL approach (PL group), and the remaining 11 by the interlobar fissure approach (IF group).

**Results:**

Patients’ characteristics did not significantly differ between the two groups. In the PL group, 34 underwent video-assisted thoracoscopic surgery (VATS), and 6 underwent robot-assisted thoracoscopic surgery. In the IF group, all 11 underwent VATS. Operation duration, estimated blood loss, and postoperative complication frequency were not significantly different between these groups, but the maximum tumor diameter showed a significant difference.

**Conclusions:**

Complete TS of the S9, S10, and both through the PL is a reasonable option for tumors located in such segments. This approach is a feasible option for performing TS.

## Background

Generally, early-stage non-small-cell lung cancer (NSCLC) is treated by surgical resection. Recently, advances in computed tomography (CT) technology have made the detection of small and peripheral lung nodules, including early-stage NSCLC, possible. Conventionally, standard procedures for small NSCLC include lobectomy combined with systematic lymph node dissection. Although segmentectomy applies only as a compromise for patients with impaired lung function, radical segmentectomy could be a viable alternative to standard lobectomy in select patients. Additionally, high-resolution CT (HRCT) can clearly detect lungs’ detailed interior structures, including the pulmonary ligaments (PLs). The PL has a double serous layer of visceral pleura forming the intersegmental septum and enters the lung parenchyma [1]. Through this clear visualization, a new surgical approach has been established. In 2011, we first reported the most technically challenging thoracoscopic segmentectomy (TS) of the lateral basal segment (S9), the posterior basal segment (S10), and both through the PL [2].

The objective of this retrospective study was to determine whether the PL approach could be a surgical option for performing TS of the S9, S10, or both and compare the efficacy of the PL and interlobar fissure (IF) approaches.

## Methods

### Study design and patients

This study retrospectively reviewed the medical records of an institution. A written informed consent was obtained from each patient. The Research Ethics Committee of the Tokyo Women’s Medical University approved this study (No.: 4988 and 5363).

Between February 2009 and November 2021, 542 patients underwent segmentectomy for malignant lung tumors at Tokyo Women’s Medical University Hospital (Tokyo, Japan). The indication criteria for segmentectomy included the presence of pure ground-glass nodule of less than 2 cm, metastatic lung tumor, or low pulmonary function. We extracted those who underwent segmentectomy of the S9, S10, and both. Ultimately, 51 patients were included. Among them, 40 underwent a complete thoracoscopic segment resection of the S9, S10, or both by the PL approach (PL group), and 11 underwent such a procedure by the IF approach (IF group). Complete TS of the S9, S10, and both using the PL approach was performed in cases where PLs were detected using preoperative CT.

We collected preoperative evaluation data such as patient’s comprehensive history, physical examination result, CT scan imaging data, positron emission tomography imaging data, and pulmonary function test results.

Resected specimens were examined histopathologically, and histological typing was performed according to the World Health Organization/International Association for the Study of Lung Cancer Histological Classification of Lung Tumors [3]. We also reclassified each case according to the eighth edition of the TNM classification for lung cancer.

Furthermore, patients’ characteristics, intra- and postoperative complications, and surgical outcomes were analyzed. The diagnosis of recurrent disease was made by radiographic and pathological confirmation. Tumor progression within the ipsilateral hilum or mediastinum indicated local recurrence.

### Preoperative surgical planning

**Fig. 1:**
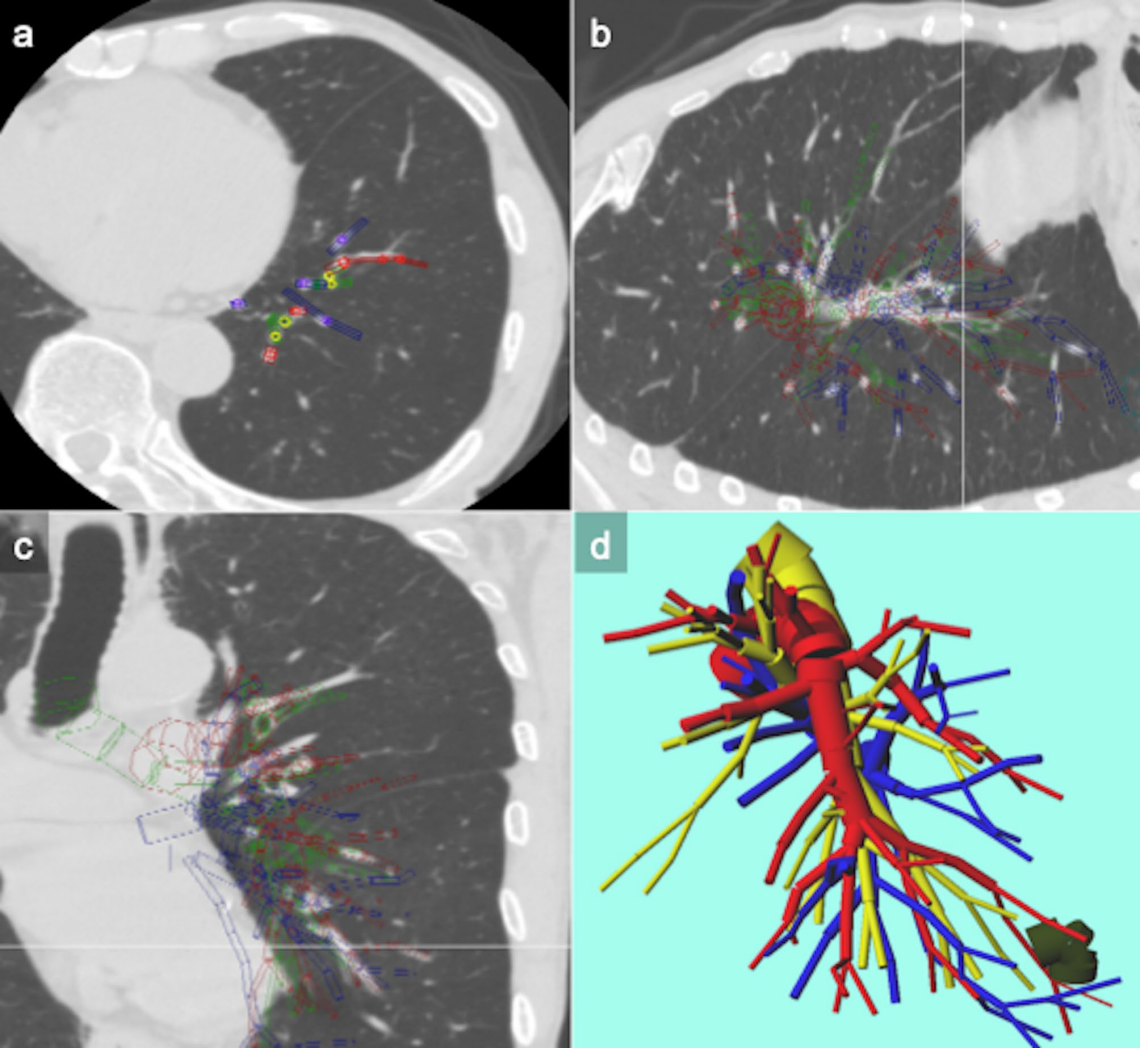
Creation of patient-actual virtual 3D pulmonary model After the communications-in-medicine (DICOM) format images, which were obtained from 1 mm thin-sliced high-resolution computed tomography (HRCT)-scan images of tumor and hilum, were uploaded to a personal computer, the homemade software “CTTRY” (Tokyo Women’s Medical University, Tokyo, Japan) allowed surgeons to mark pulmonary arteries, veins, bronchi, and tumor on the HRCT image manually and attempt to reconstruct an anatomical model with the help of anatomically correct images (A–C). The location and thickness of the pulmonary vessels and bronchi were rendered as various sizes of cylinders. In accordance with the resulting numerical data, a 3D image was reconstructed with Metasequoia shareware (http://metaseq.net/) (D)

Our department used virtual three-dimensional (3D) pulmonary reconstruction models for preoperative surgical planning. The reconstruction of individual 3D pulmonary models was previously described [4–7]. After HRCT examination, the imaging data were viewed in digital imaging and communications-in-medicine (DICOM) format. After plain chest CT slices of 1-mm thickness were obtained, the HRCT images were converted into DICOM data, which were inputted into a personal computer. The individual 3D pulmonary models were reconstructed using CITTRY software, whereas the pulmonary vessel and bronchial reconstructions were made manually and continuously drawn. The bronchi, pulmonary arteries and veins, tumor, and pulmonary lobes detected on all generated CT scans were manually traced using CTTRY on a personal computer. The location and thickness of the bronchi and pulmonary vessels were rendered as various sizes of cylinders. Based on the resulting numerical data, a 3D image, which was converted by the surface-rendering technique, was reconstructed using Metasequoia LE (https://www.metaseq.net/jp/; Tetraface Inc., Tokyo, Japan), which is a free software. The reconstructed 3D images of the pulmonary blood vessels, bronchi, and lung can be reshaped, transected, and moved even on a personal computer; thus, virtual surgical procedures and navigation during surgery can be simulated (Fig. 1) [4–7].

### Surgical procedures

Division of the intersegmental septum formed by the PL exposed the inferior pulmonary vein, and both the lateral (V9) and posterior basal (V10) veins were then transected. Next, the intersegmental septum was dissected to expose the bronchi and pulmonary arteries. The target bronchi were exposed and transected, followed by the target pulmonary arteries. Below are the surgical procedures for video-assisted thoracoscopic surgery (VATS) and robot-assisted thoracoscopic surgery (RATS) separately.

#### VATS

**Fig. 2:**
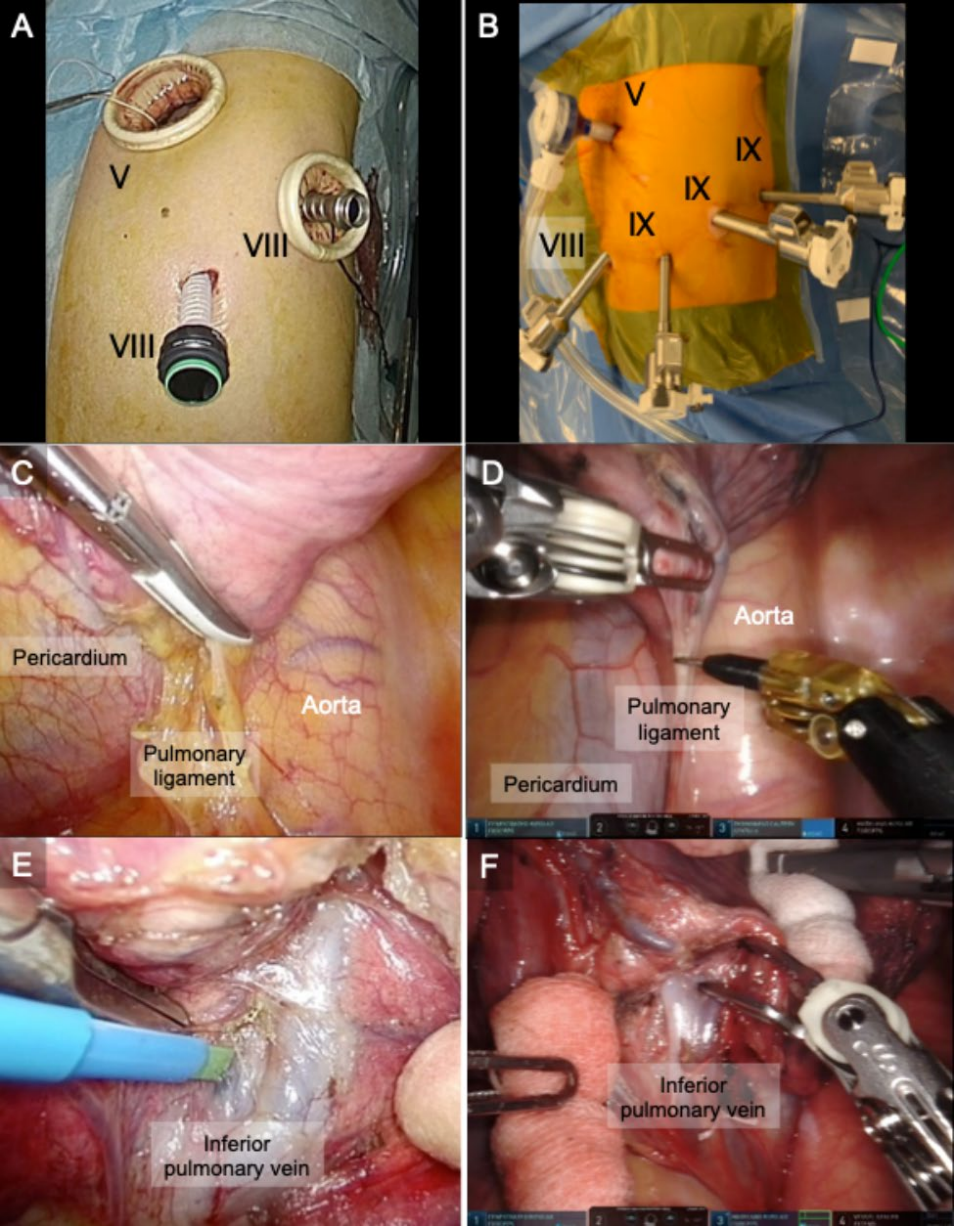
Intraoperative view of video-assisted thoracoscopic surgery (VATS) and robot-assisted thoracoscopic surgery (RATS). Left and right columns of photographs represent VATS and RATS. Port placement of VATS (A) and RATS (B) in the left chest. A small skin incision was made along the anterior-axillary line at the 5th intercostal space (ICS). A camera port was placed at the middle-axillary line of the 8th ICS, and an assist port was placed at the posterior-axillary line of the 8th ICS (A). A 12 mm trocar (AirSeal® access ports, ConMed, Utica, NY) was inserted into the 5th ICS anteriorly in the anterior-axillary line as the assistant port. A robotic camera port was placed at the middle-axillary line of the 9th ICS. Then, an 8 mm robotic trocar was inserted in the 9th ICS at the posterior side of the tip of the scapula. Two 12 mm robotic trocars were inserted in the 8th ICS anteriorly in the anterior-axillary line and the 9th ICS at the posterior-axillary line (B). After lifting the lower lobe, the pulmonary ligament (PL) was incised up to the inferior pulmonary vein in VATS (C and E) and RATS (D and F). In VATS, the forceps were manipulated from above the monitor screen (C and E), whereas in RATS, the robotic forceps were operated from the bottom of the monitor screen (D and F). The black dot line indicates the PL location

In VATS, the patient underwent general anesthesia with one-lung ventilation while lying in the lateral decubitus position. A small skin incision (2.5–4 cm long) was made along the anterior-axillary line at the fifth intercostal space (ICS). Then, a camera port and an assistant port were placed at the midaxillary line of the eighth or ninth ICS and at the posterior-axillary line of the eighth or ninth ICS, respectively (Fig. 2a). The placement was checked on two monitors using a rigid 30° endoscope. Division of the intersegmental septum formed by the PL exposed the inferior pulmonary vein and its branches, the superior (V6), and the basal vein (Fig. 2c and e). Subsequently, the intersegmental septum was dissected to expose the pulmonary arteries running alongside the bronchi, which were also transected. When the bronchi were exposed, the regional lymph nodes were dissected for intraoperative frozen-section diagnosis. After transection of the bronchi and pulmonary arteries, anterior basal vein (V8) was exposed and lung parenchyma was dissected along V8 for dividing the segments. The intersegmental plane was identified through the intersegmental veins. Especially, V6b + c was exposed after dissecting the outer sidewall of the S9 and S10 bronchi. Sufficiently exposing the V6b + c ensured the superior margin distance for S9, S10, or both. Lymph nodes were assessed during an intraoperative pathological examination. Patients with primary lung cancer underwent levels 11 and 12 with/without level 7 lymph node dissections.

#### RATS

Similar to the VATS procedure, RATS segmentectomy of the S9, S10, and both were performed using five-port incisions, with an assistant port used as a carbon dioxide (CO_2_) insufflation port. With the pleural space as the entry point, a 12 mm trocar (AirSeal® access ports, ConMed, Largo, FL, USA) was inserted as an assistant port in the fifth ICS anteriorly in the anterior-axillary line. Moreover, two 8 mm robotic trocars were inserted, one as a port for the robotic camera in the ninth ICS at the middle-axillary line and the other as port 4 in the posterior side of the scapula’s tip. In addition, two 12 mm robotic trocars were inserted in ports 1 and 3 in the eighth ICS anteriorly along the anterior-axillary line and in the ninth ICS along the posterior-axillary line, respectively. Thereafter, the da Vinci Xi® surgical system (Intuitive Surgical, Sunnyvale, CA, USA) was docked (Fig. 2b), and all of its four robotic arms were used. A CO_2_ insufflation system (AirSeal® System, ConMed) set at 5 mmHg was also utilized. The robotic instruments were manipulated through a 12 mm port mounting a reducer measuring 12–8 mm. For visualization, a robotic 30° endoscope with a camera was used. Moreover, fenestrated bipolar forceps, a permanent cautery spatula, and Cadiere forceps were inserted through ports 1, 3, and 4 (intuitive surgery), respectively. After lifting the lower lobe using the Cadiere forceps, the PL was incised up to the inferior pulmonary vein (Fig. 2d and f). After the basal pulmonary vein was exposed, both the V9 and V10 were transected using robot staplers, and V8 was exposed and lung parenchyma was dissected along V8. Next, by dissecting the intersegmental septum, the target bronchi were exposed and transected, followed by the target pulmonary arteries. After indocyanine green was injected intravenously, observation under fluorescence navigation revealed intersegmental planes, which were marked using the fenestrated bipolar forceps and permanent cautery spatula. Thereafter, the target S9, S10, or both were resected using the robot staplers. After removing the resected lung segments from the thoracic cavity, the remaining lower lobe of the lung was pulled toward the sternum. Lymph nodes at levels 7, 11, and 12 were dissected after making a mediastinal pleural incision cranially along the bronchi from the bronchial stump.

### Statistical analysis

All statistical data were analyzed using the JMP Pro software (version 16.0.0, SAS Institute, Cary, NC, USA). P-values were calculated by Fisher’s exact test or chi-square test for the categorical data and Mann–Whitney U test for the continuous data. We express continuous variables as the median with minimum and maximum values, and categorical variables as numbers and percentages (%). A P-value of less than 0.05 was considered significant.

## Results

Table 1 shows the characteristics of 51 patients. The median age of 40 patients in the PL group was 62.5 (22–82) years, and that of 11 patients in the IF group was 66.0 (52–80) years. Sex showed no significant difference between the two groups. In the preoperative pulmonary function test, the median functional vital capacity (FVC) was 3.07 (2.03–5.75) and 3.35 (2.31–5.88) L, the median forced expiratory volume in 1 s (FEV_1.0_) was 2.41 (1.30–4.34) and 2.38 (1.74–3.65) L, and the median percentage of diffusing capacity corrected for alveolar volume (%DL_CO_) was 78.8% (35.3–114.9%) and 80.1% (59.8–89.0%) in the PL and IF groups, respectively. Pulmonary function was not significantly different between the two groups.

**Table 1:** Patients’ characteristics

		PL group	IF group	*P*-value
Age, median (years)		62.5 (22–82)	66.0 (52–80)	0.47
Sex				
	Male, n (%)	14 (35.0)	6 (54.5)	0.30
	Female, n (%)	26 (65.0)	5 (45.5)	
Comorbidity				
	Hypertension, n (%)	14 (35.0)	4 (36.4)	0.73
	Diabetes mellitus, n (%)	4 (10.0)	2 (18.2)	0.60
	COPD, n (%)	5 (12.5)	2 (18.2)	0.64
	CAD, n (%)	5 (12.5)	0 (0)	0.57
	CVD, n (%)	2 (5.0)	0 (0)	1.00
	Collagen disease, n (%)	2 (5.0)	0 (0)	1.00
FVC, median (L)		3.07 (2.03–5.75)	3.35 (2.31–5.88)	0.57
FEV_1.0_, median (L)		2.41 (1.30–4.34)	2.38 (1.74–3.65)	0.95
%DL_CO_, median (L)		78.8 (35.3–114.9)	80.1 (59.8–89.0)	0.67

*P*-values were calculated by Fisher’s exact test or chi-square test for categorical data and Mann–Whitney *U* test for continuous data. Continuous variables are expressed as the median with minimum and maximum values, and categorical variables are expressed as numbers and percentages (%). PL, pulmonary ligament; IF, interlobar fissure; COPD, chronic obstructive pulmonary disease; CAD, coronary artery disease; CVD, cerebrovascular disease; FVC, functional vital capacity; FEV_1.0_, forced expiratory volume in 1 s; %DL_CO_, percentage of diffusing capacity corrected for alveolar volume.

Tables 2 and 3 list the resected segments and surgical outcomes, respectively. In the PL group, 34 patients underwent VATS, and 6 underwent RATS. In the IF group, all 11 patients underwent VATS. The median operation duration was 211 (129–404) minutes in the PL group and 184 (110–276) minutes in the IF group, and the median estimated blood loss was 14 (2–332) and 20 (5–105) mL, respectively; no significant differences were observed between the two groups. The surgery was not converted into open thoracotomy in both groups. The median chest tube duration was 4 (2–11) and 3 (3–14) days, and the median postoperative hospital stay was 7.5 (5–14) and 8.0 (5–17) days in the PL and IF groups, respectively. No intraoperative complication was noted in both groups. Postoperative complications such as air leaks that lasted more than 7 days and arrhythmias were observed in 3 patients in the PL group (7.5%) and 1 patient in the IF group (9.1%). One patient with paroxysmal atrial fibrillation in the PL group and 2 patients with prolonged air leaks in both groups were classified as Grade II by the Clavien–Dindo classification. The frequency of postoperative complications was not significantly different between the two groups. The 30- and 90-day mortality rates were 0%. None of the patients was readmitted within 30 days. The PL and IF groups differed significantly in terms of the maximum tumor diameter; the median values were 13.5 (3–21) and 19 (10–40) mm, respectively. Regarding tumor histology, 17 patients in the PL group had lung cancer (16 had adenocarcinoma, and 1 had squamous cell carcinoma) and 23 had metastatic lung tumors. In the IF group, the tumor histology was adenocarcinoma in 3 and metastatic lung tumors in 8 patients.

**Table 2:** Resected segments

		PL group	IF group
Location, n (%)			
Right	S9	3 (7.9)	1 (9.7)
	S10	4 (10.0)	1 (9.7)
	S9 + S10	6 (15.5)	1 (9.7)
Left	S9	5 (12.5)	4 (36.3)
	S10	8 (20.0)	2 (18.2)
	S9 + S10	14 (35.5)	2 (18.2)

Categorical variables are expressed as numbers and percentages (%). PL, pulmonary ligament; IF, interlobar fissure; S9, lateral basal segment; S10, posterior basal segment.

**Table 3:** Surgical outcomes

			PL group	IF group	*P*-value
Surgical procedure, n (%)					
	VATS		34 (85.0)	11 (100.0)	0.32
	RATS		6 (15.0)	0	
Conversion into open thoracotomy, n			0	0	
Operation duration, median (min)			211 (118–404)	182 (110–276)	0.13
Blood loss, median (mL)			14 (2–332)	20 (5–105)	0.29
Chest tube duration, median (days)			4 (2–11)	3 (1–14)	0.93
Postoperative hospital stay, median (days)			7.5 (5–14)	8.0 (4–17)	0.99
Intraoperative complications, n			0	0	1.00
Postoperative complications, n (%)			3 (7.5)	1 (9.1)	1.00
	Persistent air leaks, n (%)		1 (2.5)	1 (9.1)	0.39
	Atrial fibrillation, n (%)		1 (2.5)	0	1.00
	PVC, n (%)		1 (2.5)	0	1.00
30-day mortality, n			0	0	
90-day mortality, n			0	0	
Maximal tumor diameter, median (mm)			13.5 (3–25)	19.0 (10–40)	0.01
Pathology, n (%)					
	Lung cancer	Ad	16 (40.0)	3 (27.3)	0.61
		Sq	1 (2.5)	0	
	Metastases		23 (57.5)	8 (72.7)	

*P*-values were calculated by Fisher’s exact test or chi-square test for categorical data and Mann–Whitney *U* test for continuous data. Continuous variables are expressed as the median with minimum and maximum values, and categorical variables are expressed as numbers and percentages (%). PL, pulmonary ligament; IF, interlobar fissure; VATS, video-assisted thoracoscopic surgery; RATS, robot-assisted thoracoscopic surgery; PVC, premature ventricular contraction; Ad, adenocarcinoma; Sq, squamous cell carcinoma.

RATS was performed on six patients in the PL group. The median operation time was 332 (260–404) minutes and the median estimated blood loss was 9 (4–20) ml.

## Discussion

Although applied only as a compromise for patients with poor lung function and other comorbidities when formal anatomical lobectomy is contraindicated, sublobar resection became an alternative for stage IA NSCLC measuring 2 cm or less. It is technically more difficult than lobectomy, requiring intimate 3D knowledge of the relationship between the relevant bronchi and pulmonary vessels. Owing to the remarkable progress in imaging technology, 3D processing of CT images became possible. The 3D branching pattern of the intersegmental pulmonary veins can be identified using 3D images reconstructed from CT data. Previously, our department reported a patient-specific virtual 3D pulmonary model for thoracoscopic lung resections [4–7]. In fact, the pulmonary blood vessels in the 3D model created preoperatively using CTTRY were accurately depicted [8].

The key tissue in our surgical procedure is the PL. This tissue can be detected by advanced CT techniques in many patients, and it consists of a double serous layer of the visceral pleura that forms the intersegmental septum [2]. Although there are exceptions, the intersegmental septum is divided into the medial basal segment (S7) and S10 of the right lung, and anterior basal segment (S8) and S10 of the left lung [1]. The PL can easily be surgically separated from the lung parenchyma, and resection between lung segments with an automatic suture device is unnecessary. Thus, the PL is useful for intersegmental division of the lung parenchyma during segmentectomy of the S9, S10, or both [2]. The frequency of postoperative persistent air leaks was not significantly different in the PL group compared with that in the IF group. Furthermore, the PL was incised up to the inferior pulmonary vein, allowing the basal pulmonary vein to be easily exposed. Intersegmental veins were present in the intersegmental septum in all cases. In addition, the pulmonary arteries running alongside the bronchi were exposed by intersegmental septum dissection. Even if the intersegmental septum was poor, vessels and bronchi were identified by dissecting the accurate intersegmental division. Thus, interlobar separation of the lungs is not mandatory to expose the pulmonary artery.

Sublobar lung resection such as segmentectomy and subsegmentectomy has histologically been objective to extirpate with minimal loss of functioning lung tissue. Several reports have demonstrated that it can preserve lung function compared with lobectomy [9]. Thoracoscopic lung segmentectomy is rather challenging because the intersegmental border is difficult to identify and divide. However, we previously reported that 3D images reconstructed from CT data are useful for understanding the 3D branching pattern of the intersegmental pulmonary veins, allowing us to identify the intersegmental plane [4–7]. Conventional segmentectomy of the S9, S10, or both involves an interlobar fissure that cuts into the lung parenchyma between the superior segment and the S8. In our previous report, thoracoscopic partial resection of the S9, S10, or both was performed using a new approach that does not require interlobar separation, reconstructing individual 3D pulmonary models [2]. After we reported TS of the S9, S10, and both through a PL approach, several related papers have been published [10, 11]. As one of the advantages, surgical manipulation is considered easy during the ipsilateral second surgery and in the subsequent surgery after our segmentectomy because interlobar separation was not performed. Furthermore, because the postoperative complication rate was 7.5% and the 30- and 90-day mortality rates were 0% in the PL group, segmentectomy using a PL approach is considered relatively safe. Moreover, surgical outcomes in the PL approach were similar to those in the IF approach in this study. Therefore, PL approach could be a potential alternative for performing complete TS of the S9, S10, or both. On the other hand, the disadvantage of the technique is the difficulty in recognizing the vessels and bronchi. Carefully checking the individual 3D pulmonary model reconstruction is crucial for preoperatively and intraoperatively identifying the vessels and bronchi.

The operation time in the PL group was longer than that in the IF group. This difference in the operating time can be attributed to the fact that first-time segmental resections were performed by surgeons, including trainees, in the PL group; however, surgeries were performed by fully qualified surgeons in the IF group. Nevertheless, no significant difference in operation times was detected between the groups. Operation times may shorten after surgeons gain more experience in performing segmentectomy via the PL approach. Postoperative air leaks were observed while using PL approach and the chest tube duration in the PL group was slightly longer than in the IF group. However, postoperative air leaks are now decreasing and the chest tube durations in the PL group are shortening.

Oncological outcomes are important issues when performing sublobar lung resection for malignant lung tumors. According to a recent report, although local recurrence occurred more frequently with segmentectomy than with lobectomy, overall survival in patients with stage IA NSCLC measuring ≤ 2 cm was superior with segmentectomy than with lobectomy [12]. Segmentectomy preserves more lung parenchyma than lobectomy and allows for more extensive treatment for not only relapse of the primary lung cancer and a second primary lung cancer but also possibly for other cancers and lethal diseases that might be present. Therefore, overall survival was speculated to be exceeded. If the lung ligaments are clearly visible on CT, they can be easily divided into the S7 and S10 of the right lung and the S8 and S10 segments of the left lung. The intersegmental septum that constitutes the pulmonary ligament may have secured a surgical margin from the tumor in the PL group with no local recurrence. Therefore, segmentectomy using the PL approach is one of the effective surgical methods for resecting lung tumors in S9 or S10. As discussed previously, ipsilateral second surgery and subsequent postsegmentectomy surgery using a PL approach were available as options because interlobar separation was not performed.

Considering the increasing number of RATS procedures being performed in Japan, the National Health Insurance began covering robotic pulmonary segmentectomy for malignant lung tumors in 2020. In the present study, 6 patients in the PL group underwent RATS since 2020. Recently, robotic segmentectomy has been performed safely, with excellent perioperative outcomes and safety levels [13–15]. In contrast, the surgeon at the console can easily dissect the intersegmental septum to expose the pulmonary vein.

Previously, we reported that VATS segmentectomy using a PL approach involves a higher risk of incomplete lymph node dissection than conventional procedures [2]. To overcome this problem, we introduced RATS. As indicated in other studies, performing lymph node dissection is easier through RATS than VATS; moreover, RATS segmentectomy made it easy to perform lymph node dissection [16, 17]. In RATS, the target segments were removed using PL approach and lymph node dissections were performed along the bronchial stump. RATS could dissect the lymph nodes around the lobe bronchus and perform the dissection of levels 7, 11, and 12 lymph nodes routinely. Although the operation duration of RATS, which has a small number of cases, was longer than the conventional VATS in the present study, segmentectomy of the S9, S10, and both thorough the PL approach remains extremely technically challenging. Given that the RATS technique is faster to master, the reported operation duration might be shortened if the number of RATS cases increases [18, 19].

As for the important study limitations, this study is a nonrandomized and retrospective single-center study with a small sample size. A comparative study with a larger case series is needed to determine the real significance of complete TS of the S9, S10, and both through the PL approach.

## Conclusions

In conclusion, complete TS of the S9, S10, or both through the PL approach is a reasonable option for tumors located in such segments. This approach is considered a feasible option for performing TS.

## Data Availability

All the data and materials supporting our findings are included within the article.

## List of abbreviations

NSCLC: non-small cell lung cancer
CT: computed tomography
HRCT: high-resolution computed tomography
PL: pulmonary ligament
TS: thoracoscopic segmentectomy
S9: lateral basal segment
S10: posterior basal segment
3D: three-dimensional
V9: lateral basal vein
V10: posterior basal vein
VATS: video-assisted thoracoscopic surgery
RATS: robot-assisted thoracoscopic surgery
ICS: intercostal space
V6: superior pulmonary vein
V8: anterior basal vein
CO2: carbon dioxide
FVC: functional vital capacity
FEV_1.0_: forced expiratory volume in 1 s
%DL_CO_: percentage of diffusing capacity corrected for alveolar volume
S7: medial basal segment
S8: anterior basal segment
